# Cost-effectiveness of dengue vaccination in Yucatán, Mexico using a dynamic dengue transmission model

**DOI:** 10.1371/journal.pone.0175020

**Published:** 2017-04-05

**Authors:** Eunha Shim

**Affiliations:** Department of Mathematics, Soongsil University, Seoul, Republic of Korea; Universidad Nacional de la Plata, ARGENTINA

## Abstract

**Background:**

The incidence of dengue fever (DF) is steadily increasing in Mexico, burdening health systems with consequent morbidities and mortalities. On December 9^th^, 2015, Mexico became the first country for which the dengue vaccine was approved for use. In anticipation of a vaccine rollout, analysis of the cost-effectiveness of the dengue vaccination program that quantifies the dynamics of disease transmission is essential.

**Methods:**

We developed a dynamic transmission model of dengue in Yucatán, Mexico and its proposed vaccination program to incorporate herd immunity into our analysis of cost-effectiveness analysis. Our model also incorporates important characteristics of dengue epidemiology, such as clinical cross-immunity and susceptibility enhancement upon secondary infection. Using our model, we evaluated the cost-effectiveness and economic impact of an imperfect dengue vaccine in Yucatán, Mexico.

**Conclusions:**

Our study indicates that a dengue vaccination program would prevent 90% of cases of symptomatic DF incidence as well as 90% of dengue hemorrhagic fever (DHF) incidence and dengue-related deaths annually. We conclude that a dengue vaccine program in Yucatán, Mexico would be very cost-effective as long as the vaccination cost per individual is less than $140 and $214 from health care and societal perspectives, respectively. Furthermore, at an exemplary vaccination cost of $250 USD per individual on average, dengue vaccination is likely to be cost-effective 43% and 88% of the time from health care and societal perspectives, respectively.

## Introduction

Dengue fever (DF) is a febrile illness that is caused by any one of four serotypes of flavivirus (DENV-1, DENV-2, DENV-3, and DENV-4) that cross-react immunologically. Dengue is endemic in more than 100 countries, causing more than 390 million infections annually, 96 million of which are clinical dengue infections [1–4]. Infection with dengue virus provides serotype-specific, long-term protection as well as temporary cross-protection against the other serotypes for 6–24 months [3, 5]. However, people who have had a single primary infection have a higher risk of developing dengue hemorrhagic fever (DHF) and dengue shock syndrome (DSS) upon a second infection, a phenomenon attributed to antibody-dependent enhancement (ADE) [3, 6]. Thus, more dengue diseases occur primarily in patients who reside in hyperendemic areas in which multiple serotypes circulate simultaneously. Although mild dengue disease and DF contribute to more than half of the total public health burden of dengue-associated illnesses [7], the more serious manifestations of DHF and DSS are the major impetuses behind emerging efforts to prevent infection [8].

Currently, there is no direct therapy available that works against the dengue virus, and thus, the only means of dengue control are vaccination and various forms of vector control. In Mexico, the aggregate economic cost of dengue was $170 (95% CI $151-$292) million, or $1.56 (95% CI: $1.38-$2.68) per capita, including both direct and indirect costs associated with dengue [9]. Specifically, the cost associated with hospitalized patients was $25 million, whereas the ambulatory and fatal episodes of dengue represent $54 million and $8 million per year, respectively [9]. In addition, surveillance and vector control cost represents 48.9% of the total economic burden of dengue in the country, which equates to $83 million per year [9]. Specifically, the median annual estimated expenditure for insecticide products per household that took action was $31 [10]. However, vector control has been only partially successful in reducing the dengue disease burden, which increases the importance of prevention [3, 11, 12].

In December 2015, Mexico approved the use of the world's first vaccine against dengue, Dengvaxia^®^, and became the first country in the world to approve the sale of the dengue vaccine. In Mexico, Dengvaxia^®^ will be available to children over the age of nine and adults under 45 who live in areas where the disease is endemic. The World Health Organization (WHO) Strategic Advisory Group of Experts recommended that Dengvaxia^®^ be introduced only in geographic settings with a seroprevalence ≥70% in the age group targeted for vaccination [13]. In Yucatán, Mexico, the overall seroprevalence of dengue infection is 81.5%, and the seroprevalence between ages 9 and 45 is estimated to be 82.3% [14].

Dengvaxia^®^ is a tetravalent chimeric yellow-fever dengue (CYD) vaccine that was shown to have different levels of efficacy against the four serotypes, with its highest efficacy against serotypes 3 and 4. In a phase III randomized, controlled vaccine trials of CYD-TDV conducted in Latin American countries, the overall efficacy of the vaccine against virologically confirmed dengue cases was 64.7% (95% CI [58.7, 69.8]) [15, 16]. Furthermore, the vaccine efficacy was dependent on individuals’ serostatus, ranging from 52.5% among seronegative individuals (95% CI [5.9, 76.1]) to 81.9% among seropositive individuals (95% CI [67.2, 90.0]) [16–18]. In addition, analysis of the phase III trials of CYD-TDV indicated that an increased risk of severe disease is associated with breakthrough dengue infections in vaccinated seronegative individuals [19].

To examine the impact of a partially effective dengue vaccine, we developed a mathematical model of dengue transmission and vaccination. As the international use of the tetravalent dengue vaccine might be approved in the near future, evaluation of the potential impact that the dengue vaccine might have at the population level will be critical for optimizing public health strategies. There have been previous studies that have estimated the economic impact of dengue vaccines in Asia and South America, including Thailand, Argentina, and Brazil [20–24], however, to our knowledge, ours is the first analysis of the cost-effectiveness of a dengue vaccination in Mexico with consideration of ADE. Whereas previous modeling studies have investigated the epidemiological effects of vaccine-induced ADE on dengue spread [25–28], its impact on the cost-effectiveness of dengue vaccination in Mexico remains unknown. In prior analyses, it was indicated that the epidemiological impact of the vaccine depends strongly on the local transmission intensity and the degree of vaccine-induced ADE, which could result in hospitalization for individuals who are vaccinated when they are seronegative and contract dengue disease thereafter [26, 27].

In this paper, we developed a dynamic age-structured model of dengue transmission to examine the cost-effectiveness of dengue vaccination programs, taking into account the enhanced susceptibility upon secondary infection and the role of clinical cross-immunity as well as vaccine-induced ADE. Using a currently adopted vaccination strategy against dengue (i.e., targeting individuals aged 9–45) in Yucatán, Mexico, we evaluated the potential reduction of overall dengue incidence as well as the cost-effectiveness of dengue vaccination programs from both health care and societal perspectives. We found that the threshold vaccination cost per individual at which dengue vaccination becomes very cost-effective is $205 and $310 from health care and societal perspectives, respectively. Furthermore, we assessed the incremental cost-effectiveness ratio (ICER) in terms of dollars per quality-adjusted life year (QALY) gained for a range of vaccine costs. We also performed a probabilistic sensitivity analysis to present cost-effectiveness acceptability. Although it is shown that a dengue vaccine program in Yucatán, Mexico would be very cost-effective at a range of vaccine pricing, our probabilistic sensitivity analysis indicates that the cost-effectiveness of dengue vaccination could be affected by various parameters, and thus epidemiological impact of a dengue vaccination program needs to be carefully examined before providing guidance on dengue vaccination based on modeling results.

## Materials and methods

### Model description

**Fig 1:**
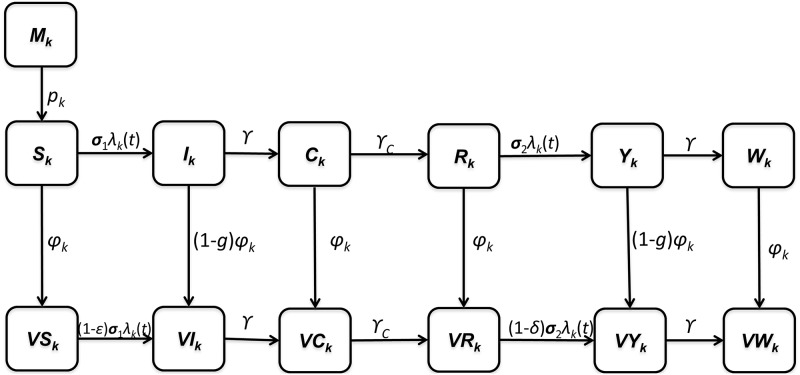
Diagram of the age-structured model used for analyses. Populations are divided into dengue-related, age-dependent epidemiological classes. The subscript *k* indicates these age groups (*k* = 1, …, 11). The age groups used for the model were ages <1, 1–4, 5–8, 9–14, 15–19, 20–24, 25–45, 46–49, 50–59, 60–64, and over 65.

To describe the spread of the dengue virus, we developed an age-structured model that considers both primary and secondary infections (Fig 1). Our model considers both asymptomatic infections and symptomatic infections. The proportion, *g*_*x*_, of infected individuals is assumed to be symptomatic, where the subscript *x* refers to the epidemiological status of individuals (Table 1). Symptomatic infections are further separated into mild or severe cases in which the probability of severe disease is assumed to be dependent on past infection history and vaccination status, consistent with empirical data (Tables A and B in S1 Appendix) [23, 29, 30]. Specifically, individuals with secondary infections are assumed to be at a higher risk of experiencing DHF and DSS than those with primary infections. Therefore, our model considers both clinical cross-immunity and the role of ADE, i.e., differential risks of developing severe cases upon primary and secondary infection [29, 31]. Furthermore, in our model, an individual recovering from a secondary infection is assumed to become immune to all serotypes because third or fourth infections from dengue are very rare [30, 32]. As in our model, a two-serotype dengue model would be satisfactory in Mexico, given that a relatively steady two-serotype dominant state exists [33]. In fact, all four dengue serotypes have been observed in Mexico, but DENV-1 have been the predominant serotype since 2008, with DENV-2 representing most of the remaining cases (17% in 2009 and 16% in 2010) [18, 33].

**Table 1:** Epidemiological parameters.

Symbol	Parameter	Value	Distribution	Refs
*f*_*k*_	Fertility rate in age group *k*	*f*_*1*_ = *f*_*2*_ = *f*_*3*_ = *f*_*4*_ = *f*_*9*_ = *f*_*10*_ = *f*_*11*_ = 0, *f*_*5*_ = 0.0904 x 10^−3^, *f*_*6*_ = 0.1732 x 10^−3^, *f*_*7*_ = 0.0903 x 10^−3^, *f*_*8*_ = 0.0025 x 10^−3^	Point estimate	[34]
*N*_*k*_	Relative size of age group *k*	*N*_*1*_ = 0.0190, *N*_*2*_ = 0.0760, *N*_*3*_ = 0.0797, *N*_*4*_ = 0.1185,*N*_*5*_ = 0.0993, *N*_*6*_ = 0.0892, *N*_*7*_ = 0.2935, *N*_*8*_ = 0.0534, *N*_*9*_ = 0.0808, *N*_*10*_ = 0.0281,*N*_*11*_ = 0.0625	Point estimate	[34]
*B*	Birth rate in Mexico, $b=\sum_{k=1}^{11}f_{k}N_{k}$.	4.8348 x 10^−5^	Point estimate	Modeling assumption
*p*_*k*_	Rate of aging out of age group *k*	*p*_*1*_ = 0.0027, *p*_*2*_ = 0.0007, *p*_*3*_ = 0.0005, *p*_*4*_ = 0.0005 *p*_*5*_ = 0.0005, *p*_*6*_ = 0.0005, *p*_*7*_ = 0.0001, *p*_*8*_ = 0.0005, *p*_*9*_ = 0.0003, *p*_*10*_ = 0.0005, *p*_*11*_ = 0.0001	Point estimate	Modeling assumption (*p*_*k*_ = 1/*a*_*k*_ where *a*_*k*_ is the age interval in age group *k*)
*μ*_*k*_	Death rate in age group *k*	*μ*_*1*_ = *b*/*N*1—*p*_1_*μ*_*k*_ = *p*_*k*-1_*N*_*k*-1_/*N*_*k*_*—p*_*k*_ (*k≠*1)	Point estimate	[35]
*σ_n_*	Relative probability of being susceptible to the *n*^th^ infection	(5-*n*)/4	Point estimate	[23]
*β*_*0*_	Base probability of transmission rate	*β*_*0*_	Triangular(0.3969*β*_*0*_, *β*_*0*_, 2.1272*β*_*0*_)	[36]
*ϕ*_*k*_	Vaccination rate in age group *k*	Varies	Point estimate	Modeling assumption
*ε*	Vaccine efficacy against infection among seronegative individuals aged nine and over	0.616	Triangular(0.351, 0.616, 0.881)	[17]
*δ*	Vaccine efficacy against infection among seropositive individuals aged nine and over	0.792	Triangular(0.472, 0.792, 0.927)	[17]
*g*_*x*_	Proportion of dengue infections that are symptomatic in the epidemiological class *x*_*k*_	0.45 for *x*_*k*_ = *I*_*k*_ 0.8 for *x*_*k*_ = *Y*_*k*_ or *VI*_*k*_ 0.14 for *x*_*k*_ = *VY*_*k*_	Point estimate	[26, 37, 38]
*y*	Fraction of DF cases that sought medical care	0.5	Triangular(0.4, 0.5, 0.6)	[39, 40]
*γ*	Rate of recovery from infection	0.146 day^-1^	Point estimate	[31]
*γ*_*C*_	Rate of loss of cross-immunity	0.0055 day^-1^	Beta (37.3, 6790)	[41, 42]
*h*_*x*_	Probability of developing DHF/DSS after symptomatic infection among the individuals in the epidemiological class *x*_*k*_	0.045 for *x*_*k*_ = *Y*_*k*_ or *VI*_*k*_ 0.25*h*_*Y*_ for *x*_*k*_ = *I*_*k*_, *J*_*k*_ or *VY*_*k*_	Point estimate	[26]
*χ*	Risk of death from DHF/DSS	0.01	Beta (2, 198)	[2, 23]

These parameters and values were used in the analyses unless indicated otherwise.

It is known that, soon after birth, infants are protected against dengue by high levels of maternal antibodies [43]. Thus, in our model, the population contains a resistant infant class of children under the age of one with maternal antibodies (*M*_1_) that wanes at rate *p*_1_. In addition, the population contains 10 other distinct age classes that represent individuals aged 1–4, 5–8, 9–14, 15–19, 20–24, 25–45, 46–49, 50–59, 60–64, and over 65. Transition rates among these age classes are independent of infection status and occur through aging at rate *p*_*k*_ (*k* = 1, …, *11*). Within each age class, we incorporated 13 epidemiological classes. Detailed descriptions of these epidemiological classes can be found in Table 2. Infected individuals are assumed to recover from primary infections at rate *γ* and gain clinical cross-protection, which prevents clinical illness but allows sero-conversion. The average duration of clinical cross-protection is assumed to be 1/*γ*_*C*_ (Table 1). Individuals who recover from secondary infections (*W*^*k*^ and *VW*^*k*^) are assumed to be immune to all strains. The rates of birth and death are denoted by *b* and *μ*_*k*_, respectively.

**Table 2:** Model variables. The subscript *k* refers to the age group *k*.

Symbol	Variable
*M*_*k*_	Number of individuals protected by maternal antibodies in age group *k* (*M*_*k*_ = 0 for *k*≠1)
*S*_*k*_	Number of susceptible unvaccinated individuals in age group *k*
*I*_*k*_	Number of primarily infected unvaccinated individuals in age group *k*
*C*_*k*_	Number of unvaccinated individuals recovering from primary infections who are temporarily protected against clinical disease in age group *k*
*R*_*k*_	Number of unvaccinated individuals susceptible to secondary infections in age group *k*
*Y*_*k*_	Number of unvaccinated individuals with secondary infections in age group *k*
*W*_*k*_	Number of unvaccinated individuals recovering from secondary infections in age group *k*
*VS*_*k*_	Number of partially susceptible vaccinated individuals in age group *k*
*VI*_*k*_	Number of primarily infected vaccinated individuals in age group *k*
*VC*_*k*_	Number of vaccinated individuals recovering from primary infections and temporarily protected against clinical disease in age group *k*
*VR*_*k*_	Number of vaccinated individuals susceptible to secondary infections in age group *k*
*VY*_*k*_	Number of vaccinated individuals with secondary infections in age group *k*
*VW*_*k*_	Number of vaccinated individuals recovering from secondary infections in age group *k*

Our vaccination strategy is implemented by vaccinating individuals aged 9–45, consistent with a current recommendation in Mexico for the dengue vaccine. Specifically, for age groups *k* = 3, 4, 5, and 6, individuals except those who are symptomatically infected are assumed to be vaccinated at the rate of *ϕ*_*k*_. We assumed that the vaccine efficacy was consistent with the findings from the phase III trial results for CYD-TDV in Latin America [15, 16] (Table 2). That is, prior dengue infection was shown to increase vaccine efficacy. Thus, our model assumes that the dengue vaccine efficacy is lower among seronegative individuals than seropositive ones (*ε* < *ϕ*) (Table 2) [15, 16, 44, 45]. Furthermore, our model incorporates vaccine-enhanced dengue disease among the vaccinated, as observed in the CYD-TDV trials [15, 46]. Specifically, we defined *h*_*I*_ and *h*_*VI*_ as the probability of developing DHF among symptomatically infected individuals in *I*_*k*_ and *VI*_*k*_, respectively, and assumed that the probability of developing DHF/DSS after primary symptomatic infection among individuals who were seronegative when vaccinated would be higher than among unvaccinated individuals (*h*_*VI*_ > *h*_*I*_) (Table 2).

Using these notations and assumptions, the age-structured model of dengue transmission and vaccination is given by:

$$\begin{array}{ll} \frac{dM_{1}}{dt} & =b-(p_{1}+\mu_{1})M_{1},\\ \frac{dS_{k}}{dt} & =p_{k-1}S_{k-1}+\Phi_{k}M_{k-1}-(\varphi_{k}+\lambda_{k}+\mu_{k}+p_{k})S_{k},\\ \frac{dI_{k}}{dt} & =p_{k-1}I_{k-1}+\lambda_{k}S_{k}-[\gamma +(1-g)\varphi_{k}+\mu_{k}+p_{k}]I_{k},\\ \frac{dC_{k}}{dt} & =p_{k-1}C_{k-1}+\gamma I_{k}-[\gamma_{C}+\varphi_{k}+\mu_{k}+p_{k}]C_{k},\\ \frac{dR_{k}}{dt} & =p_{k-1}R_{k-1}+\gamma_{C}C_{k}-[\lambda_{k}+\varphi_{k}+\mu_{k}+p_{k}]R_{k},\\ \frac{dY_{k}}{dt} & =p_{k-1}Y_{k-1}+\lambda_{k}R_{k}-[\gamma +(1-g)\varphi_{k}+\mu_{k}+p_{k}]Y_{k},\\ \frac{dW_{k}}{dt} & =p_{k-1}W_{k-1}+\gamma Y_{k}-[\varphi_{k}+\mu_{k}+p_{k}]W_{k},\\ \frac{dVS_{k}}{dt} & =p_{k-1}VS_{k-1}+\varphi_{k}S_{k}-[(1-\varepsilon )\lambda_{k}+\mu_{k}+p_{k}]VS_{k},\\ \frac{dVI_{k}}{dt} & =p_{k-1}VI_{k-1}+(1-g)\varphi_{k}I_{k}+(1-\varepsilon )\lambda_{k}VS_{k}-[\gamma +\mu_{k}+p_{k}]VI_{k},\\ \frac{dVC_{k}}{dt} & =p_{k-1}VC_{k-1}+\varphi_{k}C_{k}+\gamma VI_{k}-[\gamma_{C}+\mu_{k}+p_{k}]VC_{k}, \end{array}$$

$$\begin{array}{ll} \frac{dVR_{k}}{dt} & =p_{k-1}VR_{k-1}+\varphi_{k}R_{k}+\gamma_{C}VC_{k}-[(1-\delta )\lambda_{k}+\mu_{k}+p_{k}]VR_{k},\\ \frac{dVY_{k}}{dt} & =p_{k-1}VY_{k-1}+(1-g)\varphi_{k}Y_{k}+(1-\delta )\lambda_{k}VR_{k}-[\gamma +\mu_{k}+p_{k}]VY_{k},\\ \frac{dVW_{k}}{dt} & =p_{k-1}VW_{k-1}+\varphi_{k}W_{k}+\gamma VY_{k}-[\mu_{k}+p_{k}]VW_{k} \end{array}$$

where $b=\sum_{k=1}^{11}f_{k}N_{k}$, $\lambda_{k}=\beta_{k}\sum_{k=1}^{11}\left(I_{k}+VI_{k}+Y_{k}+VY_{k}\right)/N$, and $\Phi_{k}=\{\begin{array}{ll} p_{1}, & k=2\\ 0, & \text{otherwise}. \end{array}$Here, $\frac{dM_{k}}{dt}=0$(*k* = 2, …, 11) and $\frac{dS_{1}}{dt}=\frac{dI_{1}}{dt}=\frac{dC_{1}}{dt}=\frac{dR_{1}}{dt}=\frac{dY_{1}}{dt}=\frac{dW_{1}}{dt}=\frac{dVS_{1}}{dt}=\frac{dVI_{1}}{dt}=\frac{dVC_{1}}{dt}=\frac{dVR_{1}}{dt}=\frac{dVY_{1}}{dt}=\frac{dVW_{1}}{dt}=0$.

### Calibration

Using baseline parameters, we ran the model to equilibrium. To generate country-specific epidemiological DHF profiles, we allowed the transmission rates to be age-dependent. Specifically, we defined *β*_*i*_ as the relative susceptibility of age group *k*. These rates were chosen to capture the patterns of empirical dengue incidence in Yucatán, Mexico [14, 33]. Age-specific incidence profiles were obtained by using *β*_1_ = 0, *β*_2_ = 0.0877, *β*_3_ = 0.1349, *β*_4_ = 0.3507, *β*_5_ = 0.3237, *β*_6_ = 0.2158, *β*_7_ = 0.0809, *β*_8_ = 0.2158, *β*_9_ = 0.1754, *β*_10_ = 0.5396, and *β*_11_ = 0.2428. Once *β*_*k*_ values were set, the base probability of transmission rate (*β*_*0*_) was set such that the annual incidence of symptomatic dengue infection in Mexico is 539 per 100,000, comparable with empirical estimates [9, 36, 47]. The annual incidence of DHF in Mexico is estimated to be 12 per 100,000 with adjustments that account for underreporting [36, 48, 49]. Vaccination rates were chosen such that the potential target vaccine coverage, stated as 30% of the eligible individuals, would be reached [50].

For a probabilistic sensitivity analysis, we conducted a Monte Carlo sampling that gave us a distribution of realistic dengue incidence over time for the analysis. This distribution of dengue incidence was generated by repeatedly sampling from parameter distributions (Table 1). Our probabilistic sensitivity analysis generated a range of feasible predictions of dengue incidence by varying the base probability of transmission rate (*β*_*0*_) and the costs associated with infection and vaccination from which cost-effectiveness were calculated. Furthermore, at various levels of willingness to pay, the cost-effectiveness acceptability was determined. In supplementary materials, probabilistic sensitivity analyses using lower and higher than 30% of vaccine coverage levels are also presented.

### Direct medical, non-medical, and indirect unit costs

As is customary in analyses of cost-effectiveness, all outcomes (health and economic) were discounted at a uniform rate of 3% per year, and all costs were standardized to 2016 USD using the consumer price index [51]. Direct medical costs were calculated based on an average cost estimate per bed-day and per outpatient visit [9]. Combining these data with the distribution of cases, we derived an average cost estimate per DF and per DHF (i.e., *C*_*DF*, *direct*_ and *C*_*DHF*, *direct*_, respectively). Non-medical direct costs were obtained from prior studies based on patient interviews [9]. We estimated indirect costs based on productivity losses from the number of school days and workdays lost. The estimated average daily unit costs for elementary education (5–14 year olds) and high school education (15–18 year olds) were $8.80 and $10.60, respectively [9]. For children, the average school days lost due to dengue episodes were 6.2 days per hospitalized case and 4.4 days per ambulatory case. A workday lost for economically active and non-economically active adults was estimated to be $12.82/day and $4.89/day, respectively [9]. Overall, the economic value of the average workday lost was $9.53 [9]. For adults, the average workdays lost due to dengue episodes were 9.8 days per hospitalized case and 5.4 days per ambulatory case. In addition, we included the indirect costs of household members affected by inpatients or outpatients (Table B in S1 Appendix).

### Calculation of Disability-Adjusted Life Years (DALYs) and costs associated with dengue

Analyses were performed from both the health care perspective (only direct medical costs) and the societal perspective (direct medical, direct non-medical, and indirect costs). We used disability weights for DF and DHF to calculate the quality-adjusted life-years (QALYs) lost in association with dengue episodes. A disability weight of 1 was used for premature death. QALYs lost by each case were calculated using the following equation [22, 52]:

$$QALY(D,L,a_{k})=-\frac{DCe^{-ha_{k}}}{{\left(h+r\right)}^{2}}\left[e^{-(h+r)L}\{1+(h+r)(L+a_{k})\}-\{1+a_{k}(h+r)\}\right]$$

where *D* is the disability weight (*D* = *D*_*DF*,_ *D*_*DHF*,_ or *D*_*Death*_); *C* is the age-weighting correction constant; *h* is the parameter from the age-weighting function; *a*_*k*_ is the average age of the individual in the age group *k*; *L* is the duration of the disability or the years of life lost due to premature death expressed in years (*L* = *L*_*DF*,_ *L*_*DHF*,_ or *L*_*Death*,*k*_); and *r* is the social discount rate. The age-weighting function represents the value of life at different ages, giving a higher weight to the healthy life years lived between the ages of 9 and 54, as this period of life is considered to be socially more important than the younger and older years of life [52].

The rate of new DF cases, DHF cases, and deaths in age group *k* are calculated as following:

$$\begin{array}{ll} \frac{dDF_{k}(t)}{dt} & =g_{I}(1-h_{I})\sigma_{1}\lambda_{k}S_{k}+g_{Y}(1-h_{Y})\sigma_{2}\lambda_{k}R_{k}\\ & +(1-\varepsilon )g_{VI}(1-h_{VI})\sigma_{1}\lambda_{k}VS_{k}+(1-\delta )g_{VY}(1-h_{VY})\sigma_{2}\lambda_{k}VR_{k},\\ \frac{dDHF_{k}(t)}{dt} & =g_{I}h_{I}\sigma_{1}\lambda_{k}S_{k}+g_{Y}h_{Y}\sigma_{2}\lambda_{k}R_{k}+(1-\varepsilon )g_{VI}h_{VI}\sigma_{1}\lambda_{k}VS_{k}+(1-\delta )g_{VY}h_{VY}\sigma_{2}\lambda_{k}VR_{k},\\ \frac{dDeath_{k}(t)}{dt} & =\chi \frac{dDHF_{k}(t)}{dt} \end{array}$$

Costs = Costs of vaccination + Costs associated with dengue infection (DH and DHF)

$$=\int_{0}^{T_{f}}\left\{\begin{array}{l} \sum_{k=2}^{11}C_{V,k}\phi_{k}\left(S_{k}+(1-g_{I})I_{k}+C_{k}+R_{k}+(1-g_{Y})Y_{k}+W_{k}\right)\\ +\sum_{k=1}^{11}\left(C_{DF,k}\frac{dDF_{k}(t)}{dt}+C_{DHF,k}\frac{dDHF_{k}(t)}{dt}\right) \end{array}\right\}{\left(1+r\right)}^{-t/365}dt$$

Total QALYs lost = $\int_{0}^{T_{f}}\sum_{k=1}^{11}\left[\begin{array}{l} \Delta QALY(D_{DF},L_{DF},k)\left(\frac{dDF_{k}(t)}{dt}\right)\\ +\Delta QALY(D_{DHF},L_{DHF},k)\left(\frac{dDHF_{k}(t)}{dt}-\frac{dDeath_{k}(t)}{dt}\right)\\ +\Delta QALY(D_{Death},L_{Death},k)\left(\frac{dDeath_{k}(t)}{dt}\right) \end{array}\right]dt$

## Results

We calculated the equilibria for the annual dengue infection incidence in the absence of vaccination (1,060 per 100,000) and the annual symptomatic dengue fever incidence (539 per 100,000), which were comparable to empirical estimates [36, 37, 47–49]. The annual DHF incidence in Yucatán, Mexico was estimated to be 12 per 100,000 [48, 49]. Accordingly, we estimated the total cost of dengue per year in Yucatán, Mexico to be $1.86 million for the health care system and $3.41 million for society.

For vaccination scenarios, we assumed that vaccines were given to individuals of the target ages (i.e., ages 9–45 years old). As baseline parameter values, vaccination rates were chosen such that 30% vaccine coverage level of the vaccine-eligible population (i.e., individuals aged 9–45 years old) would be reached over 20 years, resulting in vaccination of 375,447 individuals in total. With these vaccination rates, the median cumulative simulated number of symptomatic cases was 1,116 in the population of 2 million people or 56 per 100,000 per year. After taking into account the estimated probability of seeking medical care for non-severe cases, we forecasted that 29 dengue cases would require medical care per 100,000 individuals per year. Our analysis indicates that dengue vaccination in Yucatán, Mexico would prevent 90% of symptomatic dengue fever cases as well as 90% of DHF incidence and dengue-related deaths per year, resulting in a savings of $33 million in dengue-related medical costs over 20 years. In our model, the predicted reduction in dengue incidence can be explained by both direct and indirect effects of vaccination, which are captured in a dynamic transmission model. Due to indirect effects of vaccination, the percentage of reduction of the dengue incidence is likely to be higher than the percentage of the vaccine coverage, consistent with prior studies [53, 54]. When a critical portion of the population is vaccinated against the dengue virus, a majority of community members are protected by direct and/or indirect effects of vaccination.

**Fig 2:**
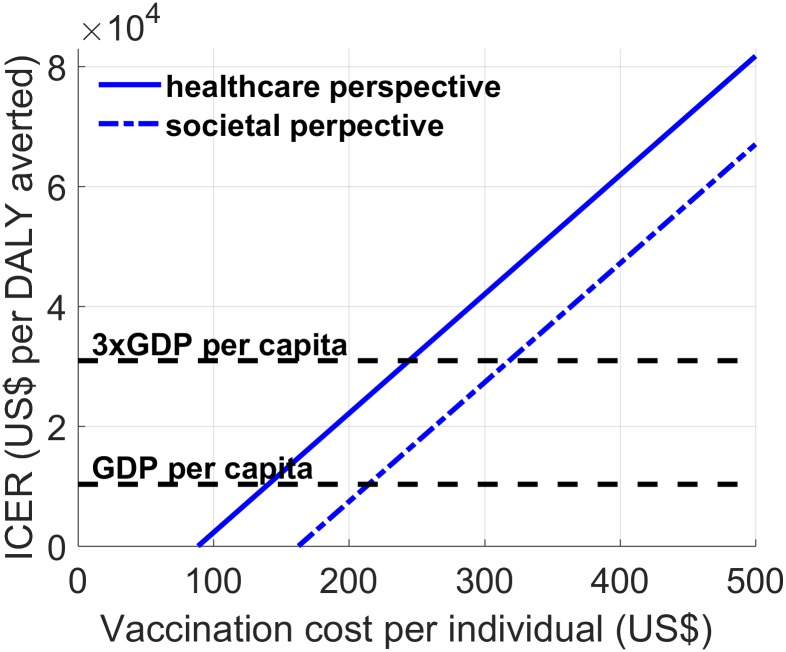
Cost-effectiveness of dengue vaccines. Cost per DALY averted for dengue vaccination programs. GDP indicates the gross domestic product per capita, and 3x GDP indicates costs that are thrice those of the GDP. A comparison between the costs per DALY averted for the vaccination program and the cost-effectiveness threshold (GDP and 3x GDP) is presented. When the costs per DALY averted are lower than the GDP per capita, the vaccination program is very cost-effective. When the costs per DALY averted are between the GDP per capita and thrice that of the GDP per capita, the vaccination program is cost-effective. If the costs per DALY averted are higher than thrice that of the GDP per capita, the vaccination program is not considered cost-effective in Yucatán, Mexico.

We evaluated the cost-effectiveness of the vaccine program with increasing vaccination costs per individual (Fig 2). For low vaccination costs, vaccines presented net savings per DALY averted (avoided costs were greater than vaccination costs); thus, they were very cost-effective. For instance, at a vaccination cost of $89 or lower, dengue vaccination would result in a net cost-savings from both the health care and the societal perspectives. For instance, at a vaccination cost of $80 per person, a dengue vaccination program in Yucatán, Mexico would generate net savings of $3.25 million from the health care perspective and $31.05 million from the societal perspective over the 20 year forecast period.

We found that the break-even total cost of dengue vaccination (where net savings equals net costs) is $89 per complete course of vaccination from the health care perspective and $163 per course of vaccination from the societal perspective (Fig 2). The cost-effectiveness ratio is negative for vaccination costs below the break-even price, indicating that the total costs associated with dengue infections are substantially reduced after dengue vaccination is introduced. At a vaccination cost of $140 or lower, the cost per QALY gained from the health care perspective is less than the $10,307 gross domestic product (GDP) per capita in Mexico, making the dengue vaccination program a very cost-effective intervention. From the societal perspective, dengue vaccination is considered very cost-effective when the vaccination cost of $214 or lower. The threshold price per course of vaccination for which the cost of vaccination equaled 3 x GDP per capita is $245 from a health care perspective and $315 from a societal perspective (Fig 2). Therefore, dengue vaccination would be cost-effective when the cost of vaccination per individual is <$245 and <$315 from health care and societal perspectives, respectively.

**Fig 3:**
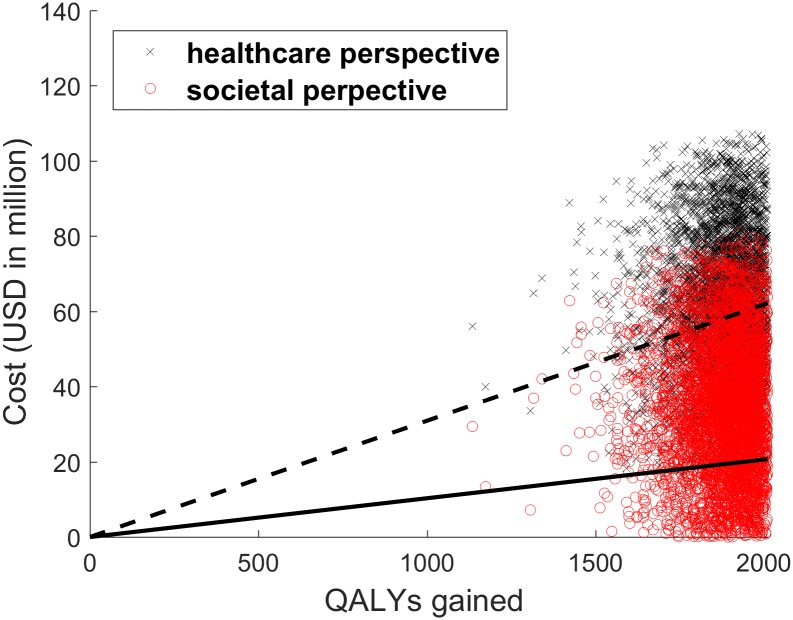
Association between discounted QALYs gained and costs of vaccination. A total of 5,000 Monte Carlo simulations of incremental cost-effectiveness ratios were plotted on a cost-effectiveness plane to compare vaccination versus no vaccination. The circles indicate the societal perspective, and crosses indicate the health care perspective. Probabilistic sensitivity analysis results are demonstrated on cost-effective planes, confirming that dengue vaccination dominates no vaccination from both societal and health care perspectives. Regions below the dashed lines indicate cost-effective (<$30,921/QALY), whereas regions below the solid lines indicate very cost-effective (<$10,307/QALY). Distribution of the cost of vaccination per individual is assumed to be Triangle ($125, $250, $375).

**Fig 4:**
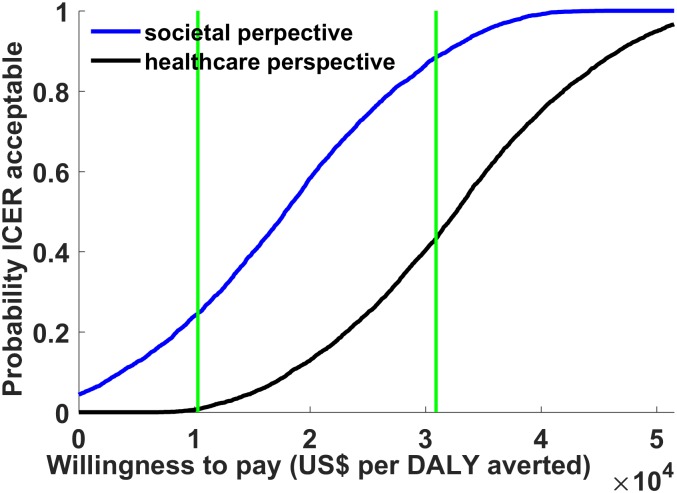
Cost-effectiveness acceptability curves. The curves show that dengue vaccination in Mexico is cost-effective at different cost-effectiveness threshold values. The cost-effectiveness acceptability curves from healthcare and societal perspectives are shown when the cost of vaccination is based on the triangle distribution with a mean value of $250, i.e., Triangle (125, 250, 375).

To evaluate parameter uncertainties, we conducted 5,000 Monte Carlo simulations for probabilistic sensitivity analyses from the healthcare and societal perspectives (Fig 3). Distributions associated with input parameters are shown in Table 1. In light of possible variations in the pricing of dengue vaccines, a distribution of the costs of vaccination per individual (Triangle (125, 250, 375)) was applied with the mean value of $250, and the results were subsequently presented in a cost-effectiveness acceptability curve (Fig 4). In this analysis, dengue vaccination was considered cost-effective 43% of the time from a health care perspective. Furthermore, dengue vaccination was cost-effective 88% of the time from a societal perspective, with a median incremental cost-effectiveness ratio of $17,878/QALY gained (95% CI $535/QALY–$35,103/QALY). This probability, however, drops to 25% when the threshold of $10,307 GDP per capita in Mexico is used, indicating that the dengue vaccination has 25% probability to be *very* cost-effective from a societal perspective. On the other hand, from a health care perspective, dengue vaccination was considered *very* cost-effective 1% of the time.

In order to examine how our results change with different vaccine coverage levels, we performed 5,000 Monte Carlo simulations for probabilistic sensitivity analyses with various vaccine coverage levels (20% and 40%) (Figures A and B in S1 Appendix). In this analysis, the probability of cost-effectiveness is likely to be higher with lower vaccine coverage than with higher vaccine coverage, as long as a relatively low threshold is used such as $10,307 GDP per capita in Mexico. However, when a higher threshold value (i.e. 3 x GDP per capita) is used, the probability of cost-effectiveness increases with vaccine coverage levels. This is because a dengue vaccination program with a relatively high vaccine coverage level result in significant reduction in dengue incidence, but due to increased cost of vaccination, its cost-effectiveness is likely to be sensitive to the willingness-to-pay value.

## Discussion

Dengue is a major public health issue in tropical and subtropical countries in Latin America and Asia. On December 9^th^, 2015, Mexico became the first country to approve a vaccine against dengue, Dengvaxia^®^[55]. Dengvaxia^®^ is the first vaccine to be licensed in the world for the prevention of dengue, and it was approved in Mexico for the prevention of dengue disease in individuals aged 9–45 years old living in endemic areas [55]. The approval of the dengue vaccine will be a critical addition to the ongoing public education and vector control efforts in Mexico, and it will help to achieve the WHO’s goal to reduce dengue mortality and morbidity by 50% and 25%, respectively, by 2020 in endemic countries [56].

In our study, we used a dynamic model to evaluate the cost-effectiveness and the economic impact of the introduction of a dengue vaccine in in Yucatán, Mexico. The results from our modelling study indicate that Dengvaxia has the potential to reduce the burden of dengue disease even in the presence of vaccine-induced ADE, potentially preventing 90% of symptomatic dengue fever cases. The greatest impact of vaccination was predicted with a relatively the highest vaccine coverage level considered (40%); however its likelihood of cost-effectiveness was shown to be sensitive to willingness-to-pay value. Thus, when a relatively low threshold value (i.e. GDP per capita) was used as willingness-to-pay value, the probability of cost-effectiveness decreased with vaccine coverage levels. Therefore, decisions at the country level for vaccine introduction should take into account affordability, and capacity for introduction as well as predicted impact and cost-effectiveness.

We also performed a probabilistic sensitivity analysis to explore how key parameters impact the probability of cost-effectiveness and to show the uncertainty in our modeling outcomes. We found that the cost-effectiveness of dengue vaccination is critically dependent on the full-course price, although dengue vaccination is likely to be cost-effective and possibly even cost saving. The threshold price at which the vaccine becomes cost-effective is of critical importance in analyses of cost-effectiveness, especially when there is no currently agreed upon price for mass vaccination, which is the case for dengue vaccination in Mexico. In middle-income countries such as Mexico, health-care budgets are lower than those of high-income nations, but governments do not benefit from charitable assistance provided to low-income countries. Mexico is not eligible for support from the Global Alliance for Vaccines and Immunization (GAVI), the nonprofit vaccines alliance, and thus the affordability of dengue vaccination remains an issue.

In light of the pricing of dengue vaccine, our threshold analysis identifies that the prices at which dengue vaccination becomes very cost-effective are $140 and $214 from health care and societal perspectives, respectively. Furthermore, it was shown that the break-even total cost of dengue vaccination (where net savings equals net costs) is $89 per complete course of vaccination. However, it was suggested that, even for higher vaccine prices, conforming the dengue vaccination to a risk stratification system could be cost-effective in predetermined high-risk regions [24]. Furthermore, vaccination of 9–45 year old individuals was shown to potentially reduce the dengue incidence significantly, potentially resulting in 90% reduction in dengue disease burden.

Our analysis also provides insights into the potential value of vaccination even in the presence of vaccine-induced ADE. Our model incorporates the risk of vaccine-induced ADE by assuming that the probability of developing DHF/DSS after a primary symptomatic infection among individuals who were seronegative when vaccinated would be higher than among unvaccinated individuals. Even with such an increased risk of DHF/DSS among vaccinated individuals, vaccination against dengue was shown to be cost-effective at a range of vaccination pricings. Overall, our results suggest that even such a vaccine with potential vaccine-induced ADE may be cost-effective and possibly cost-saving, provided that the total vaccination cost can be kept sufficiently low, below $140 for cost-effectiveness and below $89 for cost-saving.

It should be noted that the assumptions of dengue vaccination are variable in modeling studies. Thus, mathematical models of dengue transmission and vaccination vary significantly, particularly relative to the role of ADE, the incorporation of permanent cross-protection or enhancement of infection, and transient cross-protection [42]. For instance, some of the dengue transmission models, including ours, assume that disease enhancement (ADE) occurs in secondary infected hosts ([57, 58]), whereas others ([59–61]) assume that transmission enhancement occurs instead, and a few models ([29, 62]) include both effects. Assumptions for cross-protection vary between dengue models as well. Temporary cross-protection is assumed for most models, including ours ([26–29, 58, 60, 62, 63]), whereas a few models do not assume temporary cross-protection [57, 61]. Although our model incorporates key assumptions that are consistent with other modeling studies and empirical data on dengue transmission and vaccination, some of our assumptions could be revised in future work when our knowledge of dengue epidemiology and vaccine mechanisms are improved via dialogue between theoreticians and empirical researchers. For instance, studies to estimate various aspects of transmission, such as household studies, cluster targeted transmission studies, and large-scale longitudinal studies could greatly improve our knowledge of dengue transmission. Future expansions of our model could also investigate the effect of waning vaccine immunity on population protection, as the quantification of the tolerable range of ADE to maintain vaccination cost-effectiveness would be valuable information. When such data on many aspects of transmission and vaccine mechanism become available, the improved model would enable us to better guide dengue interventions. Furthermore, considering all four dengue serotypes (DENV-1, DENV-2, DENV-3, and DENV-4) would further improve the estimates on the cost-effectiveness of dengue vaccination. In addition to the mechanism of dengue transmission, future studies might also consider the impact of dengue vaccine on the prevalence of the zika virus because cross-reacting antibodies to dengue virus were recently reported to promote ADE in zika virus infections [64]. Given the cross-reaction of antibodies to dengue virus and zika virus, ADE of ZIKV infections need to be considered in vaccine approaches in the future [64].

To date, only a handful of modeling studies have been published on the cost-effectiveness of vaccination against dengue in Asia and South America [20–24]. One of these studies used a dynamic transmission model [23], whereas others used static models that did not take into account any indirect protection conferred by vaccination. Our analysis of cost-effectiveness used a dynamic transmission model that implicitly calculates the level of indirect protection afforded by mass vaccination. Thus, our model predicts a lower incidence of disease not only for vaccinated individuals, but also for those not vaccinated. This prediction leads to a larger overall reduction in dengue incidence and therefore a more favorable prediction for the cost-effectiveness of dengue vaccination. This is the first cost-effectiveness analysis of dengue vaccination in in Yucatán, Mexico. We examined the potential impact of dengue vaccination on reducing the incidence of dengue infection by simulating an age-dependent dynamic model of dengue transmission with vaccination. Our work can inform policymakers of the price at which dengue vaccination becomes cost-effective and reveals that dengue vaccination has the potential to significantly reduce the number of severe infections and dengue-related costs. Thus, introducing a dengue vaccine in in Yucatán, Mexico can effectively reduce the health and economic burdens associated with dengue infections.

## Supporting information

S1 Appendix Supplementary figures and tables. (DOC)
