# Supplementary material for: Cost-effectiveness of dengue vaccination in Yucatán, Mexico using a dynamic dengue transmission model
Source: PLoS One. 2017 Apr 5;12(4):e0175020. doi: 10.1371/journal.pone.0175020 (PMC5381893; doi:10.1371/journal.pone.0175020)
Supplement: S1 Appendix — (DOC) [file pone.0175020.s001.doc]

**Supplementary Figures and Tables**

**Figure A.** Cost-effectiveness acceptability curve showing the probability that vaccination program is cost-effective when 20% vaccine coverage level of the vaccine-eligible population (i.e., individuals aged 9-45 years old) would be reached over 20 years.

**
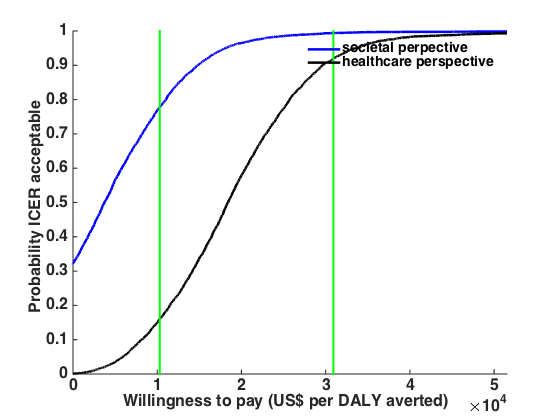
**

**Figure B.** Cost-effectiveness acceptability curve showing the probability that vaccination program is cost-effective when 40% vaccine coverage level of the vaccine-eligible population (i.e., individuals aged 9-45 years old) would be reached over 20 years

**
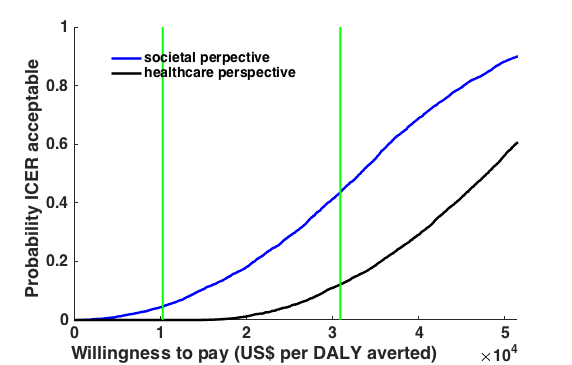
**

**Table A. Cost-effectiveness parameters.**

| **Symbol** | **Parameter** | **Value** | **Distribution** | **References** |
| --- | --- | --- | --- | --- |
| *r* | Social discount rate for DALYs calculations | 0.03 | Point estimate | [1, 2] |
| *C* | Age-weighting correction constant | 0.16243 | Point estimate | [1, 2] |
| *h* | Parameter of the age-weighting function | 0.04 | Point estimate | [1, 2] |
| *D_Death_* | Disability weight  For death | 1 | Point estimate | [1, 2] |
| *D_DF_* | Disability weight for DF | 0.197 | Beta (19.7, 80.3) | [3, 4] |
| *D_DHF_* | Disability weight for DHF/DSS | 0.545 | Beta (54.5, 45.5) | [3, 4] |
| *L_DF_* | Time lost due to DF (years) | 0.019 | Beta (5.7, 294.3) | [3, 5-7] |
| *L_DHF_* | Time lost due to DHF/DSS (years) | 0.0325 | Beta (13, 387) | [3, 6] |
| *L_Death,k_* | Years of life lost due to death for age group *k*  *k* = 2  *k* = 3  *k* = 4  *k* = 5  *k* = 6  *k* = 7  *k* = 8  *k* = 9  *k* = 10  *k* = 11 | 74.5  69.5  64.5  59.5  54.5  42  29.5  22  14.5  6 | Point estimate | Modeling assumption |
| *a_k_* | Average age of dengue exposure in age class *k*  *k* = 2  *k* = 3  *k* = 4  *k* = 5  *k* = 6  *k* = 7  *k* = 8  *k* = 9  *k* = 10  *k* = 11 | 2.5  7.5  12.5  17.5  25  35  47.5  55  62.5  71 | Point estimate | Modeling assumption |

**Table B. Probabilities and costs of dengue infections.**

|  | **Probability** | **Relative probability** | **Direct Medical costs ($)** | **Direct non-medical costs ($)** | **Indirect costs ($)**  **ages 5-14/ 15-18/ ≥19** | **Refs** |
| --- | --- | --- | --- | --- | --- | --- |
| Dengue infection in the epidemiological class *x_k_*  Asymptomatic  Symptomatic  DF  Ambulatory  Severe (DHF)  Hospitalized  Death | 1.00  1 –*g_x_*  *g_x_* | 1.00  1 - *h_x_*  *y*(1 – *h_x_*)  *h_x_*  (1- *χ*) *h_x_*  *χ h_x_* | $293  $1,171  NA | $107  $202  NA | $127/135/139  $174/185/213  $73,981 for all ages | [8]  [8]  [3, 9, 10]  [11-13]  [3, 9, 10]  [3, 13, 14]  [3, 13, 14] |
| Vaccination | Varies | | Varies | | |  |

All values are shown in 2016 US Dollars.

**References**

1. Murray CJ. Quantifying the burden of disease: the technical basis for disability-adjusted life years. Bull World Health Organ. 1994;72(3):429-45. PubMed PMID: 8062401; PubMed Central PMCID: PMCPMC2486718.

2. Carrasco LR, Lee LK, Lee VJ, Ooi EE, Shepard DS, Thein TL, et al. Economic impact of dengue illness and the cost-effectiveness of future vaccination programs in Singapore. PLoS neglected tropical diseases. 2011;5(12):e1426. doi: 10.1371/journal.pntd.0001426. PubMed PMID: 22206028; PubMed Central PMCID: PMCPMC3243704.

3. Durham DP, Ndeffo Mbah ML, Medlock J, Luz PM, Meyers LA, Paltiel AD, et al. Dengue dynamics and vaccine cost-effectiveness in Brazil. Vaccine. 2013;31(37):3957-61. Epub 2013/06/25. doi: 10.1016/j.vaccine.2013.06.036. PubMed PMID: 23791696; PubMed Central PMCID: PMC3755607.

4. Dantes HG, Farfan-Ale JA, Sarti E. Epidemiological trends of dengue disease in Mexico (2000-2011): a systematic literature search and analysis. PLoS neglected tropical diseases. 2014;8(11):e3158. doi: 10.1371/journal.pntd.0003158. PubMed PMID: 25375162; PubMed Central PMCID: PMC4222737.

5. Chao DL, Halstead SB, Halloran ME, Longini IM, Jr. Controlling dengue with vaccines in Thailand. PLoS neglected tropical diseases. 2012;6(10):e1876. Epub 2012/11/13. doi: 10.1371/journal.pntd.0001876. PubMed PMID: 23145197; PubMed Central PMCID: PMC3493390.

6. Rodriguez-Barraquer I, Mier-y-Teran-Romero L, Schwartz IB, Burke DS, Cummings DA. Potential opportunities and perils of imperfect dengue vaccines. Vaccine. 2014;32(4):514-20. doi: 10.1016/j.vaccine.2013.11.020. PubMed PMID: 24269318; PubMed Central PMCID: PMC4142437.

7. Anderson KB, Chunsuttiwat S, Nisalak A, Mammen MP, Libraty DH, Rothman AL, et al. Burden of symptomatic dengue infection in children at primary school in Thailand: a prospective study. Lancet. 2007;369(9571):1452-9. Epub 2007/05/01. doi: 10.1016/S0140-6736(07)60671-0. PubMed PMID: 17467515.

8. Bhatt S, Gething PW, Brady OJ, Messina JP, Farlow AW, Moyes CL, et al. The global distribution and burden of dengue. Nature. 2013;496(7446):504-7. doi: 10.1038/nature12060. PubMed PMID: 23563266; PubMed Central PMCID: PMC3651993.

9. Nagao Y, Koelle K. Decreases in dengue transmission may act to increase the incidence of dengue hemorrhagic fever. Proceedings of the National Academy of Sciences of the United States of America. 2008;105(6):2238-43. Epub 2008/02/06. doi: 10.1073/pnas.0709029105. PubMed PMID: 18250338; PubMed Central PMCID: PMC2538904.

10. Halstead SB. Neutralization and antibody-dependent enhancement of dengue viruses. Advances in virus research. 2003;60:421-67. Epub 2003/12/24. PubMed PMID: 14689700.

11. Shepard DS, Suaya JA, Halstead SB, Nathan MB, Gubler DJ, Mahoney RT, et al. Cost-effectiveness of a pediatric dengue vaccine. Vaccine. 2004;22(9-10):1275-80. Epub 2004/03/09. doi: 10.1016/j.vaccine.2003.09.019. PubMed PMID: 15003657.

12. Shepard DS, Coudeville L, Halasa YA, Zambrano B, Dayan GH. Economic impact of dengue illness in the Americas. The American journal of tropical medicine and hygiene. 2011;84(2):200-7. doi: 10.4269/ajtmh.2011.10-0503. PubMed PMID: 21292885; PubMed Central PMCID: PMC3029168.

13. Undurraga EA, Betancourt-Cravioto M, Ramos-Castaneda J, Martinez-Vega R, Mendez-Galvan J, Gubler DJ, et al. Economic and disease burden of dengue in Mexico. PLoS neglected tropical diseases. 2015;9(3):e0003547. doi: 10.1371/journal.pntd.0003547. PubMed PMID: 25786225; PubMed Central PMCID: PMC4364886.

14. Gubler DJ. Dengue and dengue hemorrhagic fever. Clin Microbiol Rev. 1998;11(3):480-96. PubMed PMID: 9665979.
